# Use of Eltrombopag in Children With Chronic Immune Thrombocytopenia (ITP): A Real Life Retrospective Multicenter Experience of the Italian Association of Pediatric Hematology and Oncology (AIEOP)

**DOI:** 10.3389/fmed.2020.00066

**Published:** 2020-02-28

**Authors:** Paola Giordano, Giuseppe Lassandro, Angelica Barone, Simone Cesaro, Ilaria Fotzi, Fiorina Giona, Saverio Ladogana, Maurizio Miano, Antonio Marzollo, Margherita Nardi, Lucia Dora Notarangelo, Andrea Pession, Antonio Ruggiero, Giovanna Russo, Paola Saracco, Marco Spinelli, Alessandra Tolva, Assunta Tornesello, Valentina Palladino, Giovanni Carlo Del Vecchio

**Affiliations:** ^1^Pediatric Unit, Department of Biomedical Science and Human Oncology, University of Bari “Aldo Moro”, Bari, Italy; ^2^Department of Pediatric Onco-Hematology, University Hospital of Parma, Parma, Italy; ^3^Pediatric Hematology Oncology, Department of Mother and Child, Azienda Ospedaliera Universitaria Integrata, Verona, Italy; ^4^Department Pediatric Hematology Oncology, Azienda Ospedaliero Universitaria A. Meyer Children Hospital, Florence, Italy; ^5^Department of Translational and Precision Medicine, Sapienza University of Rome, Rome, Italy; ^6^Department of Hematology, IRCCS Casa Sollievo della Sofferenza, San Giovanni Rotondo, Italy; ^7^Clinical and Experimental Hematology Unit, “G. Gaslini” Children's Hospital, Genoa, Italy; ^8^Pediatric Hematology-Oncology Unit, Department of Women's and Children's Health, Azienda Ospedaliera-University of Padova, Padua, Italy; ^9^Pediatric Hematology, Azienda Ospedaliero Universitaria Pisana, Pisa, Italy; ^10^Hemato-Oncology Unit, Children Hospital, Spedali Civili, Brescia, Italy; ^11^Department of Pediatrics, Sant'Orsola-Malpighi Hospital, University of Bologna, Bologna, Italy; ^12^Pediatric Oncology Unit, Fondazione Policlinico Universitario Gemelli IRCCS, Università Cattolica del Sacro Cuore, Rome, Italy; ^13^Pediatric Hemato-Oncology Unit, Department of Clinical and Experimental Medicine, University of Catania, Catania, Italy; ^14^Pediatric Hematology, Department of Pediatrics, University Hospital Città della Salute e della Scienza, Turin, Italy; ^15^Hemato-Oncology Unit, Fondazione MBBM, San Gerardo Hospital, Monza, Italy; ^16^Pediatric Hematology/Oncology, IRCCS Policlinico San Matteo Foundation, University of Pavia, Pavia, Italy; ^17^Pediatric Hematology Oncology, Presidio Ospedaliero Vito Fazzi, Lecce, Italy

**Keywords:** eltrombopag, children, immune thrombocytopenia, thrombopoietin receptor agonists, bleeding disorders

## Abstract

**Background:** The thrombopoietin receptor agonist eltrombopag has been shown to be safe and effective for children with chronic immune thrombocytopenia (ITP). The aim of the present study was to characterize eltrombopag use in current clinical practice.

**Material and Methods:** This is a retrospective multicenter study conducted in 17 centers affiliated to the Italian Association of Pediatric Hematology and Oncology (AIEOP). The primary objective of the study was to determine the prevalence of eltrombopag use in Italian children affected by chronic ITP, after EMA authorization for pediatric age. The secondary objective was to assess efficacy in the first 6 months and safety during the whole period of eltrombopag treatment in current clinical practice. A total of 386 children with chronic ITP were retrospectively enrolled and eligible for analysis. Among these patients, 71 received eltrombopag.

**Results:** The prevalence of eltrombopag use was 19% (95% CI 0.15–0.23). Thirty-one patients (44%) were male and 40 patients (56%) were female. The median age at the first dose of eltrombopag was 12 years (3–17 years). The median duration of eltrombopag treatment was 11 months (1–32 months) and the median starting dose was 50 mg/day (12, 5–75 mg/day). Thirty-two patients (45%) required one or more concomitant ITP medications during the first 6 months of treatment with eltrombopag. Thirty-nine patients (55%) never required concomitant medications. Median platelet counts and proportion of patients achieving the target platelet count of at least 30 × 10^9^/L and 100 × 10^9^/L significantly increased during the first 6 months of treatment (*p* < 0.0001). Additionally, eltrombopag has been proved effective in the absence of concomitant therapies. The most common Adverse Events were headache (7%) and thrombocytosis (6%).

**Conclusion:** Our study highlighted the crucial role of eltrombopag as second line treatment in children with chronic ITP.

## Introduction

Pediatric immune thrombocytopenia (ITP) is an acquired immune mediated disorder characterized by isolated thrombocytopenia (1–3). ITP is characterized by autoreactive antibodies that bind to platelets targeting them for phagocytosis by macrophages in the spleen and liver (4, 5). Other mechanisms, including B-cell hyperreactivity, T-cell–mediated cytotoxicity and impaired platelet production, have also been demonstrated to cause ITP (6–11). Although ITP is often self-limiting, about 20–30% of children develop chronic ITP, defined as thrombocytopenia persisting longer than 12 months (3). In the chronic form, therapeutic choices are complex and focused on improving health-related quality of life (HRQoL) (12, 13) and controlling bleeding symptoms (14, 15). Corticosteroids and intravenous immunoglobulins (IVIG) are recommended as first-line treatments. If first-line therapy fails, therapeutic options for managing chronic ITP include immunosuppressive drugs (such as rituximab, mycophenolate mofetil, and sirolimus) (2, 16–21), splenectomy or, more recently, thrombopoietin receptor agonists (TPO-RAs) (22, 23).

Two TPO-RAs have been studied in pediatric patients: eltrombopag and romiplostim (24, 25). Their interaction with thrombopoietin receptor stimulates proliferation and maturation of megakaryocytes, resulting in an increase of circulating platelet count (26). The oral TPO-RAs eltrombopag was the first one authorized for treatment of pediatric ITP. In August 2015 the US Food and Drug Administration (FDA) approved eltrombopag as a treatment for children aged ≥1 year with chronic ITP and refractory to other treatments. In April 2016, the European Medicines Agency (EMA) extended the marketing authorization in the European Union (EU). Recently, romiplostim use has been approved in pediatric patients with chronic ITP. Several studies investigated efficacy and safety of eltrombopag in children with ITP. In PETIT and PETIT2, two multicenter, double-blind, placebo-controlled trials, eltrombopag administration in children with ITP lasting more than 6 months resulted in increased platelet counts, reduction in bleeding symptoms, and discontinuation of concomitant therapies. Moreover, eltrombopag proved to be safe and well-tolerated (24, 27). Recently, a multicenter retrospective study reported similar results (28). Additional data about the use of eltrombopag in pediatric ITP are limited to case reports (29, 30). To date, no studies were conducted in children outside clinical trials after EMA marketing authorization of eltrombopag.

We present our multicenter retrospective experience on eltrombopag administration in Italian children with chronic ITP, with the aim to characterize the use of this agent in clinical practice outside of randomized studies.

## Materials and Methods

This is a retrospective multicenter study conducted in 17 centers affiliated to the Italian Association of Pediatric Hematology and Oncology (AIEOP). The primary objective of the study was to determine the prevalence of eltrombopag use in Italian children affected by chronic ITP, after EMA authorization for pediatric age. The secondary objective was to assess efficacy in the first 6 months and safety during the whole period of eltrombopag treatment in current clinical practice. The sample size was determined in relation to the primary objective: considering a 95% confidence level and a 5% confidence interval, at least 384 pediatric patients with chronic ITP needed to be enrolled. Inclusion criteria were: patients with chronic ITP, aged from 1 to 17 years and 364 days, with data on the treatment available after EMA authorization and with informed consent obtained from the legally authorized representatives. Exclusion criteria were: patients with non-immune or hereditary thrombocytopenia, aged under 1 year and over 18, with data on treatment after EMA authorization or signed informed consent not available. Demographic and baseline ITP data were obtained. Data regarding eltrombopag first administration including the starting dose and platelet count were collected. Duration of treatment, dose, concurrent therapies, platelet counts follow-up, and reason for interruption were assessed. Patients were monitored closely for possible adverse effects (AEs) evaluating monthly hematology assays and clinical chemistry including liver enzyme assessments. Chronic ITP was defined using the International Consensus Guidelines as a platelet count <100 × 10^9^/L that lasts 12 months or longer (3). The baseline platelet count was the pre-treatment value closest to the first dose of eltrombopag. Platelet count response was assessed at first, third, and sixth month of treatment evaluating the median platelet count and the percentage of patients with a platelet count ≥30 × 10^9^/L and ≥100 × 10^9^/L. Thrombocytosis was defined as a platelet count ≥450 × 10^9^/L (24). Increased transaminases were described for alanine aminotransferase (ALT) and aspartate aminotransferase (AST) >3× normal value according to the previous clinical trials (24, 27).

### Statistical Methods

The Stat View program (Abacus Concepts, Berkley, CA, USA) was used for statistical analysis. Nominal data are expressed as percentage and ordinal data as medians (min and max). The Friedman Test (for ordinal data), Cochran's *Q*-test (for nominal data) were performed. *P* ≥ 0.05 were considered statistically not significant (NS).

### Ethical Considerations

Prior to their participation, the caregivers provided signed consent forms after being informed about the aim of the project as foreseen by the Italian Law on Privacy and the Safeguarding of Sensitive Data (D.Lgs n196, 2003). The project was performed in accordance with the principles of the Declaration of Helsinki. Approval by the ethics committee was not necessary for a retrospective observational study on clinical practice data.

## Results

### Demographic and Baseline Clinical Data

390 patients with chronic ITP were enrolled between April 2016 and December 2018. Four patients met the criteria for exclusion leaving 386 patients enrolled and eligible for analysis. Among 386 patients with chronic ITP, 71 patients received eltrombopag. The prevalence of eltrombopag use in pediatric patients with chronic ITP was 19% (95% CI 0.15–0.23). When we divided the enrollment period into six-month time intervals, we found no difference in the incidence of eltrombopag use in temporal subgroups (data not show); Demographic and baseline clinical data are reported in Table 1. Twelve patients (17%) referred to six different centers started eltrombopag with a platelet count ≥30 × 10^9^/L. All patients had cutaneous and/or mucous bleeding and/or poor quality of life at the start of therapy. Of these patients, nine patients (75%) started eltrombopag after various second-line treatments for ITP with poor response. At the start of eltrombopag therapy two patients (17%) were on steroid therapy and eltrombopag administration was useful for reducing and discontinuing these therapies. One patient (8%) periodically underwent intravenous immunoglobulin therapy and preferred to start eltrombopag because it improved his quality of life by reducing the number of hospitalizations.

**Table 1:** Demographic and baseline clinical data of patients treated with eltrombopag.

**Total**, n°	71
**Gender**, n° (%)	
M	31 (44%)
F	40 (56%)
**Age at onset of ITP**, years	
Median (range)	7 (1–17)
**Second-line therapies prior eltrombopag**, n° (%)	38 (54%)
Cyclosporine	1 (1%)
Mycophenolate mofetil	28 (39%)
Rituximab	15 (21%)
Sirolimus	12 (17%)
Splenectomy	2 (3%)
**ITP duration before the first dose**, years	
Median (range)	2 (1–14)
**Age at the start of eltrombopag**, years	
Median (range)	12 (3–17)
**Median starting dose|**, mg	
Median (range)	50 (12,5–75)
**Baseline platelet count**, × 10^9^/L	
Median (range)	10 (1–51)
**Patients with a platelet count of at least 30 × 10^9^/L**, n°(%)	12 (17%)

### Dosing and Discontinuation Data

The median duration of eltrombopag treatment was 11 months (1–32 months). During the first 6 months of therapy, 27 patients (38%) required one or more change in eltrombopag dose. Of these, 20 patients (83%) had at least one dose increase and seven patients (17%) had at least one dose decrease. The median highest dose achieved was 75 mg/day (50–100 mg/day). Eltrombopag was administered at the dosage of 100 mg only in two patients belonging to different centers. The pro kg/ dosage was 1.1 and 1.6 mg/kg, respectively. Seventeen/71 patients (24%) discontinued eltrombopag during the first 6 months of treatment. Fourteen/71 patients (20%) discontinued permanently eltrombopag for a constant platelet count <30 × 10^9^/L, three patients at the end of third month and 11 patients after 6 months. Seven/14 patients, who permanently discontinued eltrombopag therapy, received concomitant therapies during the period of follow-up. Temporary eltrombopag interruption occurred in four patients (6%) that experienced thrombocytosis at a dosage ranging from 25 to 75 mg/day, in absence of other ITP medications.

### Concomitant Therapies

Thirty-two patients (45%) required one or more concomitant ITP medications during the first 6 months of treatment with eltrombopag. Twenty-six (37%) patients received concomitant ITP medications at start of therapy with eltrombopag, mainly consisting of IVIG (*n* = 10) and/or corticosteroids (*n* = 15). Other therapies included cyclosporine (*n* = 1), mycophenolate mofetil (*n* = 3), rituximab (*n* = 1), and sirolimus (*n* = 2). The percentage of patients requiring other drugs in association with eltrombopag was significantly reduced during the first 6 months of treatment (*p* < 0.0001). Nine (13%) of 71 patients needed concomitant treatments at the end of the first month of treatment, nine (13%) of 68 patients at the end of the third month and six (11%) of 57 patients at the end of the 6 months. Thirty-nine (55%) of 71 patients never required concomitant medications.

### Efficacy Data

Efficacy data are summarized in Table 2. Median platelet counts and proportion of patients achieving the target platelet count of at least 30 × 10^9^/L and 100 × 10^9^/L significantly increased during the first 6 months of treatment (*p* < 0.0001). One month after eltrombopag initiation, the median platelet count was 34 × 10^9^/L (1–668 × 10^9^/L), 40 of 71 patients (56%) achieved a platelet count of at least 30 × 10^9^/L and 10 of 71 patients (14%) achieved a platelet count of at least 100 × 10^9^/L. At the end of 3 months of treatments the median platelet count was 44 × 10^9^/L (1–703 × 10^9^/L), 40 of 68 patients (56%) achieved a platelet count of at least 30 × 10^9^/L and 10 of 68 patients (15%) achieved a platelet count of at least 100 × 10^9^/L. Six months after eltrombopag initiation the median platelet count was 92 × 10^9^/L (2–863 × 10^9^/L), 39 of 57 patients (68%) achieved a platelet count of at least 30 × 10^9^/L and 25 of 57 patients (44%) achieved a platelet count of at least 100 × 10^9^/L.

**Table 2:** Efficacy data.

	**T0**	**T1**	**T3**	**T6**	***P*-value**
**Platelet count**, × 10^9^/L	10	34	42	92	<0.0001
Median (range)	(1–51)	(1–668)	(1–703)	(2–863)	
**Patients with a platelet count of at least 30 × 10**^**9**^**/L**, n°/TOT (%)	12/71 (17)	40/71 (56)	40/68 (56)	39/57 (68)	<0.0001
**Patients with a platelet count of at least 100 × 10**^**9**^**/L**, n°/ TOT (%)	0/71 (0)	10/71 (14)	10/68 (15)	25/57 (44)	<0,0001

*Median platelet count (× 10^9^/L), percentage of patients with a platelet count ≥30 × 10^9^/L, percentage of patients with a platelet count ≥100 × 10^9^/L at the start of therapy (T0); after 1 month (T1); after 3 months (T3); after 6 months (T6)*.

Moreover, we have evaluated the proportion of patients achieving a platelet counts ≥50 × 10^9^/L at least once. Forty-six patients (65%) achieved platelet counts ≥50 × 10^9^/L at least once. We also found that 40% of patients achieving a platelet counts ≥50 × 10^9^/L for at least 12 weeks.

Additionally, eltrombopag efficacy was evaluated in the absence of concomitant therapies. Thirty-nine of 71 patients (55%) never needed other treatment in association with eltrombopag. In those patients, the median platelet counts and the proportion of patients achieving the target platelet count of at least 30 × 10^9^/L and 100 × 10^9^/L significantly increased during the first 6 months of treatment (*p* < 0.0001). Efficacy data in the subgroup of patients that never required concomitant medications are summarized in Table 3. Efficacy data in the subgroup of patients that required concomitant medications are summarized in Table 4.

**Table 3:** Efficacy data in the subgroup of patients that never required concomitant medications.

	**T0**	**T1**	**T3**	**T6**	***P*-value**
**Platelet count**, × 10^9^/L	12	35	58	87	<0.0001
Median (range)	(5–49)	(1–323)	(4–703)	(5–477)	
**Patients with a platelet count of at least 30 × 10**^**9**^**/L**, n°/TOT (%)	5/39 (13)	24/39 (62)	26/37 (67)	23/32 (72)	<0.0001
**Patients with a platelet count of at least 100 × 10**^**9**^**/L**, n°/ TOT (%)	0/39 (0)	4/39 (10)	12/37 (32)	13/32 (41)	<0.0001

*Median platelet count (× 10^9^/L), percentage of patients with a platelet count ≥30 × 10^9^/L, percentage of patients with a platelet count ≥100 × 10^9^/L at the start of therapy (T0); after 1 month (T1); after 3 months (T3); after 6 months (T6)*.

**Table 4:** Efficacy data in the subgroup of patients that required concomitant medications.

	**T0**	**T1**	**T3**	**T6**	***P*-value**
**Platelet count**, × 10^9^/:L	8	21	42	100	<0.0001
Median (range)	(1–51)	(1–668)	(1–272)	(2–863)	
**Patients with a platelet count of at least 30 × 10**^**9**^**/L**, n°/TOT (%)	7/32 (22)	16/32 (50)	17/31 (55)	16/25 (64)	<0.0001
**Patients with a platelet count of at least 100 × 10**^**9**^**/L**, n°/ TOT (%)	0/32 (0)	6/32 (19)	10/31 (32)	12/25 (48)	<0.0001

*Median platelet count (× 10^9^/L), percentage of patients with a platelet count ≥30 × 10^9^/L, percentage of patients with a platelet count ≥100 × 10^9^/L at the start of therapy (T0); after 1 month (T1); after 3 months (T3); after 6 months (T6)*.

### Adverse Events

AEs were evaluated during the whole period of treatment. The most frequent AEs were headache and thrombocytosis. Five patients (7%) developed one or more events of headache at a dosage ranging from 50 to 75 mg/day with a median platelet count of 199 × 10^9^/L (85–332 × 10^9^/L). Consequently, all patient reduced the dose of eltrombopag. Four patients (6%) experienced thrombocytosis at a dosage ranging from 25 to 75 mg/day in absence of other ITP medications. In all cases thrombocytosis resolved after short-term discontinuation of eltrombopag. One patient (1%), aged 16 years, while on eltrombopag (25 mg/day, 0.4 mg/kg) underwent a transitory elevation of transaminases more than three times the normal value, in the absence of either clinical symptoms or increased direct bilirubin. Common infectious and immune causes of pediatric transaminitis were negative. The elevation of transaminases resolved after a temporary dose reduction of eltrombopag. There was one case of cerebral venous thrombosis (1%) occurred in a 15 years old patient at the dosage of 75 mg/day in a patient without thrombocytosis (platelet count of 320 × 10^9^/L) and with the presence of heterozygous factor V Leiden mutation. Other transitory AEs were oral aphthous ulcers (1%), allergic urticaria (1%), and microcytic anemia (1%).

## Discussion

In Italy eltrombopag is currently provided free of charge for children aged >1 year affected by chronic ITP and refractory to other treatments. The drug is available in different dosing tablets (25, 50, and 75 mg) and the maximum recommended dosage per day is 75 mg. For children aged ≥6 years, the recommended starting dose is 50 mg per day with dose modifications of 25 mg increments, while the starting dose for children ≤5 years is 25 mg. Once initiated, the dosage should be modulated to achieve a platelet count at least of 50 × 10^9^/L and not to exceed 200 × 10^9^/L. Moreover, the aim of eltrombopag therapy should not be to normalize platelet count. However, there are no indications regarding the platelet count recommended to start treatment (31, 32). Data on eltrombopag use in children are limited to single center experiences or studies conducted outside EMA approved indications (28–30). Recently, two “real life” retrospective Italian multicenter experiences explored the benefits of eltrombopag use in adults with chronic ITP underlying its potential role in delaying splenectomy (33, 34).

Our retrospective multicenter study conducted on a large cohort of Italian children with chronic ITP evaluates the “real life” use of eltrombopag in Italy. We observed that in clinical practice eltrombopag was used in about 20% of children with chronic ITP. No other studies that assessed the prevalence of eltrombopag administration in children were conducted according to EMA recommendations. Grace et al., in a prospective, observational, longitudinal cohort study conducted before EMA authorization by the Pediatric ITP Consortium of North America (ICON1), examined second line treatment decisions in 120 children with ITP, enrolled in 2013–2015. At enrolment, about 17% of patients were treated with eltrombopag which was selected for a perceived minor toxicity, ease of administration, and expected greater compliance in children. Moreover, 52% of patients used rituximab or other immunosuppressant drugs. However, this study involved a significant proportion of children with newly diagnosed or secondary ITP (16).

We found that the use of eltrombopag in clinical practice varied widely among different centers. Therapeutic choices including the need of concomitant therapies, dosage, and platelet count at the start of eltrombopag administration were personalized among different centers taking into account the characteristics of individual patients. In our study the median starting dose was variable, sometimes above or below the recommended dosage. Moreover, physician decisions about the median platelet count at which start therapy and the need for concomitant ITP medications at baseline were individually tailored to the patient's needs. The application of a personalized therapeutic modality in chronic ITP aims to control bleeding symptoms, minimize side effects, and improve HRQoL of children and of caregivers (12). Recently, Giordano et al. explored with a narrative approach the real emotional impact of chronic ITP revealing that parent's anxiety and restrictions on children activities and sports participation have a prominent impact on their daily life with significant social, scholastic, and relational implications (13). Eltrombopag for easy administration and limited side effects could play a role in improving HRQoL but further studies are needed (35).

In our study, dosage modifications were frequently observed. Several patients initiated eltrombopag at lower doses and a higher dose was subsequently required. Further studies are needed to determine the ideal starting dose to maximize effectiveness and minimize side effects. Interestingly, in our study eltrombopag was administered at the dosage of 100 mg (above the approved level) in two patients from different centers. We have to consider that the pro kg/dosage was relatively low, 1.1 and 1.6 mg/kg, respectively. This dose adjustment improved platelet count in the subsequent follow-up in both patients without adverse events. However, further studies are needed to evaluate new dose strategies.

Furthermore, in this study eltrombopag efficacy in the first 6 months of treatment was assessed. We extended the duration of follow-up and increased the target of platelet counts compared to previous trials (24, 27) evaluating the percentage of patients with a platelet count ≥30 × 10^9^/L and ≥100 × 10^9^/L with or without concomitant therapies. In both PETIT and PETIT2, the primary outcome was achieving a platelet count ≥50 × 10^9^/L without rescue therapy. The primary end point in PETIT was to define the percentage of patients who achieved a platelet count of ≥50 × 10^9^/L at least once while on therapy, while in PETIT2 it was to assess the ability of eltrombopag to produce sustained platelet count response (defined as platelet count of ≥50 × 10^9^/L without rescue for ≥6 weeks). The results of these trials were consistent and demonstrated that eltrombopag improves platelet counts and decrease need for other ITP-therapies (24, 27). Overall, our data support that eltrombopag is effective at increasing the platelet counts and decreasing the need for additional therapy similarly to clinical trial results. In our study the median platelet counts and the proportion of patients achieving the target platelet count of at least 30 × 10^9^/L and 100 × 10^9^/L significantly increased during treatment. Moreover, the percentage of patients requiring other drugs in association with eltrombopag significantly reduced during the first 6 months of treatment. However, 14 patients (20%) discontinued permanently this medication for a constant platelet count <30 × 10^9^/L. Despite our data are promising, further studies are needed to assess the long term efficacy of eltrombopag.

In PETIT and PETIT2, the most frequent AEs included headache, upper respiratory tract infection, nasopharyngitis, diarrhea and transient liver enzyme abnormalities. No thrombotic complications were reported in both trials. In PETIT2, cataract was described in two patients, both of whom had prior corticosteroid administration and one of whom had pre-existing lens anomaly (24, 27). In our study eltrombopag appear to be well-tolerated and have an acceptable safety profile. We observed side effects similar to previous clinical trials. However, we reported one episode of thrombotic event occurred at the dosage of 75 mg/day in a patient without thrombocytosis and with the presence of heterozygous factor V Leiden mutation. Both venous and arterial thrombotic events were described in eltrombopag adult trials, even at low or normal platelet count (36, 37). Moreover, a multicenter study conducted on the off-label use of TPO-RAs in pediatric patients noted two thrombotic events not associated with thrombocytosis in patients treated with eltrombopag with underlying risk factors for thrombosis (28). Additionally, in a pediatric retrospective study including 12 patients treated with eltrombopag, one event of deep venous thrombosis associated with an ankle fracture was reported (38). Current data suggest that thrombotic risk during eltrombopag treatment should be carefully evaluated in patients with other thrombotic risk factors. Moreover, the correlation of thrombosis with increased platelet count remain unclear.

Our study has some limitations. Because of its retrospective design we are unable to establish how eltrombopag administration influence HRQoL of pediatric patients with chronic ITP. Several individual patient's characteristics such as the bleeding risk that may influence therapeutic choices are unknown. Eltrombopag could have a promising role as second line treatment in ITP to improve HRQoL in pediatric patients (39). Moreover, further prospective studies on decision making, HRQoL, and bleeding symptoms are critically needed.

In conclusion, our study provides a cross section of the “real life” use of eltrombopag in clinical practice. Despite its recent authorization marketing in Italy, eltrombopag use is spreading widely across hematology pediatric centers. Clinicians decisions regarding platelet count at the start of therapy, dosage, and association with other therapies are widely differ and are not standardized. Eltrombopag appear effective and well-tolerated in pediatric chronic ITP but further studies are needed to evaluate new dose strategies and the real long term efficacy and toxicity of eltrombopag, after its authorization in commerce.

## Data Availability Statement

The datasets generated for this study are available on request to the corresponding author.

## Ethics Statement

Ethical approval was not provided for this study on human participants because for retrospective observational studies on drugs with on-label clinical use our local ethics committee requires only a formal communication. Written informed consent to participate in this study was provided by the participants' legal guardian/next of kin.

## Author Contributions

All authors listed have made a substantial, direct and intellectual contribution to the work, and approved it for publication.

### Conflict of Interest

The authors declare that the research was conducted in the absence of any commercial or financial relationships that could be construed as a potential conflict of interest.

## Abbreviations

ITP: Immune thrombocytopenia
AIEOP: Italian Association of Pediatric Hematology and Oncology
HRQoL: Health-related quality of life
IVIG: Intravenous immunoglobulins
TPO-Ras: Thrombopoietin receptor agonists
FDA: US Food and Drug Administration
EMA: European Medicines Agency
EU: European Union
AEs: Adverse effects
ALT: Alanine aminotransferase
AST: Aspartate aminotransferase
NS: Statistically not significant
ICON1: Pediatric ITP Consortium of North America.
